# Association between diabetes mellitus and the risk of temporomandibular disorder: A nationwide population-based study

**DOI:** 10.1371/journal.pone.0355792

**Published:** 2026-08-13

**Authors:** Tsung-Fu Chang, Ling-Yu Kung, Chi-Hsiang Chung, Dun-Yu Hsu, Tsu-Hsuan Weng, Gunng-Shinng Chen, Li Chyun Yeh, Wu-Chien Chien

**Affiliations:** 1 Division of Oral Diagnosis and Family Dentistry, Department of Dentistry, Tri-Service General Hospital, Taipei, TaiwanRepublic of China; 2 School of Dentistry, College of Oral Medicine, National Defense Medical University, Taipei, TaiwanRepublic of China; 3 School of Public Health, National Defense Medical University, Taipei, TaiwanRepublic of China; 4 Department of Medical Research, Tri-Service General Hospital, National Defense Medical University, Taipei, TaiwanRepublic of China; 5 Taiwanese Injury Prevention and Safety Promotion Association (TIPSPA), Taipei, TaiwanRepublic of China; 6 Division of Orthodontics, Pediatric Dentistry and Special Need Dentistry, Department of Dentistry, Tri-Service General Hospital, Taipei, TaiwanRepublic of China; 7 Department of Nursing, University of Kang-Ning, Taipei, TaiwanRepublic of China; 8 Graduate Institute of Life Sciences, National Defense Medical University, Taipei, TaiwanRepublic of China; 9 Graduate Institute of Medical Sciences, National Defense Medical University, Taipei, TaiwanRepublic of China; Nihon University School of Dentistry at Matsudo, JAPAN

## Abstract

This study investigated the association between temporomandibular disorder (TMD) and diabetes mellitus (DM) and the potential risk factors of TMD using data from Taiwan’s Longitudinal Generation Tracking Database. A cohort of 201,465 individuals newly diagnosed with diabetes mellitus (DM) and 805,860 matched non-DM controls was observed from 2000 to 2015. To evaluate the risk factors associated with the onset of temporomandibular disorders (TMD), Cox proportional hazards regression analysis was conducted. The Kaplan–Meier method, along with the log-rank test, was applied to assess the cumulative incidence of TMD among DM patients. Results from the Cox analysis indicated that having DM significantly increased the likelihood of developing TMD (adjusted hazard ratio: 2.543; *p* < 0.001). Furthermore, DM patients who also had rheumatoid arthritis or ankylosing spondylitis faced an even higher risk, being 4.289 and 4.021 times more likely, respectively, to develop TMD compared to those without these comorbidities (*p* < 0.001). Patients with DM had 2.543 times the probability of experiencing TMD events than patients without DM. Rheumatoid arthritis and ankylosing spondylitis were associated with the highest and second highest risk of patients with DM subsequently developing TMD. Our results provide crucial indications to prevent TMD in patients with DM and improve their quality of life.

## Introduction

Temporomandibular disorder (TMD) refers to a group of conditions involving pain and impaired function of the temporomandibular joints (TMJs), the masticatory muscles, and related anatomical components. While traditionally viewed as a localized joint issue, increasing evidence suggests that TMD is influenced by systemic factors, including chronic low-grade inflammation and metabolic dysregulation [1,2]. A previous systematic review showed that TMD has a considerable effect on oral health–related quality of life, with pain being a particularly prominent factor [3]. Key symptoms of TMD often include localized pain in the facial and preauricular regions, restricted jaw mobility, and joint sounds such as clicking or popping during mandibular motion [4]. Among adults aged 20–49, the prevalence of painful TMD has been reported to be approximately 36% [5]. This condition may also be linked to broader health issues, including psychological conditions like depression, and can negatively impact a patient's overall quality of life [4]. Although the precise cause of TMD remains uncertain, current evidence suggests it results from an interplay of genetic predisposition, environmental exposures, and psychosocial influences [6–8].

Diabetes mellitus (DM) refers to a group of metabolic disorders primarily characterized by persistent hyperglycemia [9]. Chronic hyperglycemia in DM triggers systemic inflammatory cascades, elevating pro-inflammatory cytokines such as tumor necrosis factor-alpha (TNF-α) and interleukin-6 (IL-6), which can accelerate the degradation of joint [1,10]. Most DM cases are classified into two major etiological categories. Type 1 diabetes involves a complete lack of insulin production, whereas type 2 diabetes—the more common form—arises from a combination of insulin resistance and insufficient compensatory insulin secretion [11]. Additionally, DM has been linked to various musculoskeletal complications, such as restricted joint mobility, diabetic-related arthropathy, and neuropathic pain syndromes [12]. The biological plausibility of the DM–TMD relationship is further supported by the accumulation of advanced glycation end products (AGEs) in connective tissues, which increases tissue stiffness and impairs the reparative capacity of the TMJ [1]. Furthermore, diabetic neuropathy and metabolic-related obesity may lower pain thresholds and exacerbate musculoskeletal pain syndromes, potentially increasing the risk of TMD symptoms [13,14].

Some studies suggest an association between both types of DM and TMD [15]. The prevalence of TMD among patients with DM is higher [16] and the incidence of limited joint mobility, a type of TMD, is higher in patients with type 2 DM [17]. However, most previous work has relied on cross-sectional data or smaller clinical samples, leaving the long-term temporal relationship unclear [2,18,19]. Patient cohorts with an association between DM and TMD have not been extensively studied. As far as we are aware, this study is the first to explore the association between DM and the risk of TMD utilizing a large-scale, 15-year longitudinal cohort from Taiwan’s Longitudinal Generation Tracking Database (LGTD). We hypothesized that patients with DM are at an increased risk of developing TMD due to these systemic metabolic and inflammatory influences.

## Materials and methods

### Data source

Taiwan’s National Health Insurance (NHI) program is a compulsory system that provides coverage to over 23 million individuals, accounting for more than 99% of the national population. This study utilized data from the Longitudinal Health Insurance Database, a subset of the Longitudinal Generation Tracking Database (LGTD), comprising claims records for two million randomly selected individuals—representing approximately 10% of Taiwan's population. Diagnostic coding in the LGTD has followed the International Classification of Diseases, Ninth Revision, Clinical Modification (ICD-9-CM) since 2000. The reliability and validity of LGTD data, particularly regarding diagnostic and pharmaceutical coding, have been confirmed in previous validations [20,21], and its credibility is further supported by numerous peer-reviewed publications [22–24].

To protect individual privacy, all personal identifiers within the LGTD are encrypted in accordance with the data protection policies set by Taiwan's National Health Insurance Administration under the Ministry of Health and Welfare. This study adhered to the ethical principles outlined in the Declaration of Helsinki and received approval from the Institutional Review Board of Tri-Service General Hospital. An official written waiver of informed consent was granted for this research (IRB approval number: TSGHIRB No: E202416041). Consent to participate and publication were not applicable as the data were de-identified.

### Definition of diabetes mellitus

Participants were classified as having DM if they had at least three consecutive outpatient records with diagnostic codes 250.XX, 357.2, 362.0, or 366.41, or at least one inpatient record with these codes documented in the LGTD between 2000 and 2015. To ensure validity, only patients diagnosed by doctors specializing in metabolism were included.

### Study subjects and comparison groups

The study population included individuals diagnosed with both DM and TMD, identified by at least three consecutive outpatient diagnostic codes or a single inpatient record between 2000 and 2015. The control group comprised individuals without DM, randomly selected and matched to DM patients at a 1:4 ratio based on age and sex. TMD diagnoses were tracked until the individual’s death or the end of 2015. Participants who had been diagnosed with TMD prior to the initial DM diagnosis or, for controls, before their assigned index date, were excluded. For comparison subjects, the index date was defined as the date nearest to the first recorded DM diagnosis in their matched case.

### Risk variables

This study examined various demographic and contextual variables, including age, sex, insurance premium level, season of visit, geographic location, degree of urbanization, and healthcare facility level. Age was grouped into the following intervals: 0–20, 21–40, 41–50, 51–60, 61–80, and over 80 years. These age categorizations were used to maintain consistency with previous large-scale studies using Taiwan’s National Health Insurance (NHI) data and to provide clear, interpretable risk estimates for different life stages for clinical practitioners. Insurance premiums were categorized into three tiers: less than NTD 18,000, between NTD 18,000 and NTD 34,999, and NTD 35,000 or more. Geographic regions were divided into Northern, Central, Southern, and Eastern Taiwan, along with offshore islands. Urbanization levels were classified into four tiers based on population size and indicators reflecting urban development. Healthcare facility levels were grouped as local hospitals, regional hospitals, and medical centers.

Furthermore, this study evaluated the impact of various comorbid conditions, including systemic diseases [25,26] and autoimmune disorders [27]. The conditions analyzed encompassed hypertension, ischemic heart disease, chronic obstructive pulmonary disease (COPD), chronic renal insufficiency, rheumatoid arthritis, depression, ankylosing spondylitis, tinnitus, lower back pain, obstructive sleep apnea, systemic lupus erythematosus, Sjögren’s syndrome, systemic sclerosis, dermatomyositis, polymyositis, alopecia areata, ulcerative colitis, biliary cirrhosis, and autoimmune thyroid disorders, as previously reported in research related to diabetes mellitus. Corresponding ICD-9-CM codes for these diagnoses are provided in S1 Table. All comorbidities were identified based on clinical records within one year prior to the initial diagnosis of DM.

### Statistical analysis

This study employed four statistical methods for analysis. Continuous variables were compared using Student’s t-test, while categorical variables were analyzed with Pearson’s chi-square test to examine differences between the DM group and the control group. To evaluate the association between DM and the risk of developing TMD, multivariate Cox proportional hazards regression was used, generating hazard ratios (HRs) and corresponding 95% confidence intervals (CIs). The selection of covariates for our models followed an explicit causal framework (S1 Fig. in the Supporting Information), distinguishing between potential confounders (e.g., sex, age, and urbanization) and potential mediators, such as rheumatoid arthritis and ankylosing spondylitis. To ensure the validity of the Cox model, the proportional hazards assumption was verified with the Schoenfeld residual test and visual inspection of scaled Schoenfeld residual plots (S2 Fig). Data integrity aspect, given that the LGTD is a national administrative claims database where demographic and diagnostic data are mandatory for reimbursement, the core variables were highly complete. Consequently, no specific missing data handling or imputation was necessary for the variables analyzed. Data preprocessing involved cleaning and verifying the diagnostic codes to ensure the accuracy of the study and control cohorts. Multicollinearity among covariates was assessed using the variance inflation factor; variables demonstrating high collinearity, such as geographic location and urbanization level, were managed by adjusting the model variables to ensure stability. The cumulative incidence of TMD among individuals with and without DM was further assessed using the Kaplan–Meier survival analysis and log-rank test. Furthermore, we performed Fine & Gray’s sub-distribution hazard model for sensitivity test to calculate sub-distribution hazard ratios (sHRs). A p-value below 0.05 was considered statistically significant. All analyses were conducted using SPSS software, version 22 (SPSS Inc., Chicago, IL, USA).

## Results

**Fig 1:**
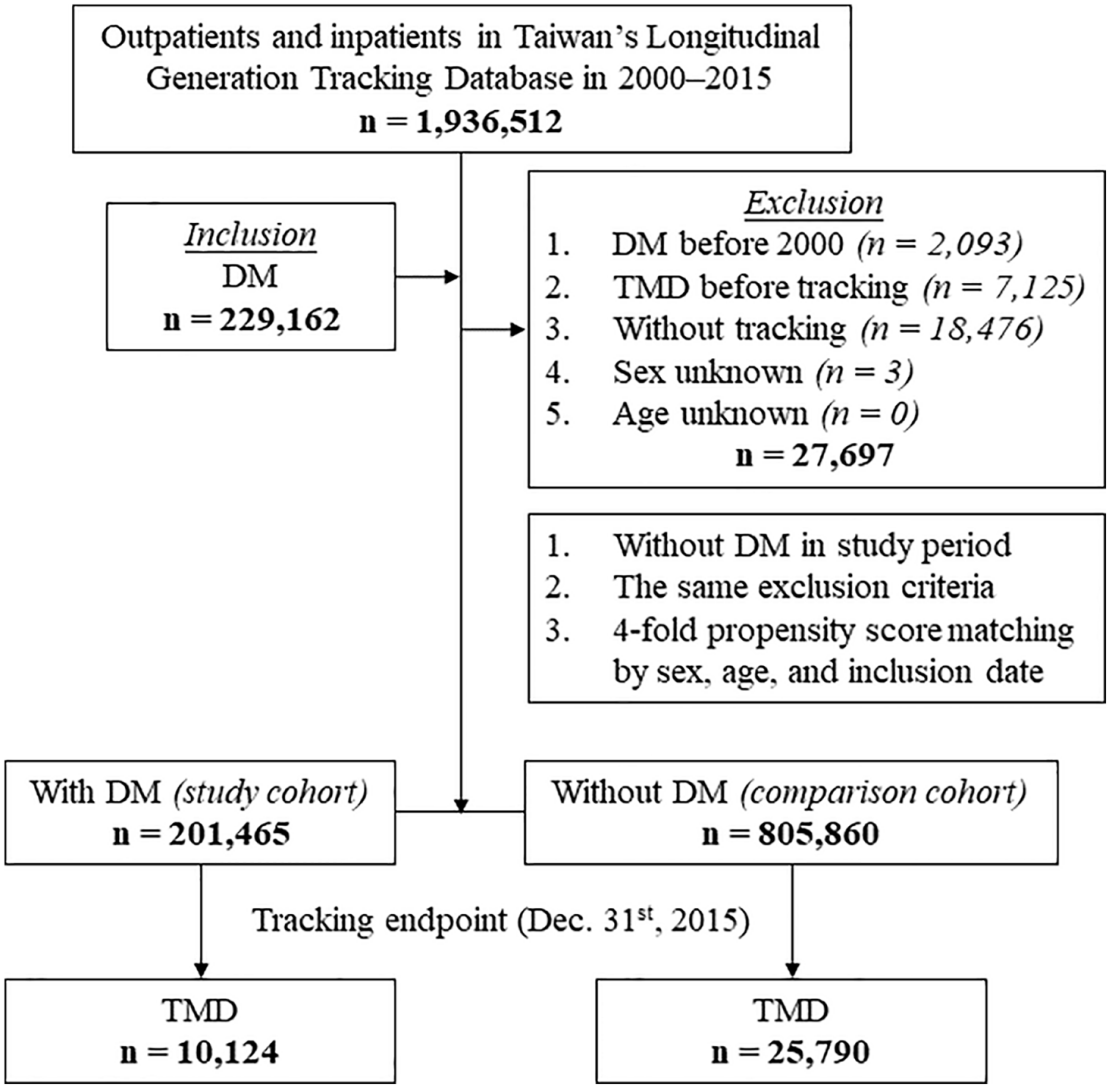
Flowchart of study sample selection from Taiwan’s Longitudinal Generation Tracking Database.

Fig 1 illustrates the participant selection flow, detailing both the inclusion and exclusion criteria, along with follow-up outcomes and the cumulative incidence of TMD in individuals with and without DM. From 2000 to 2015, after applying the exclusion criteria, the final cohort comprised 201,465 DM patients and 805,860 matched controls. The incidence of TMD in the DM group was 5.03% (10,024 out of 201,465), corresponding to 1,962.70 cases per 100,000 person-years. In comparison, the control group showed a TMD incidence of 3.2% (25,790 out of 805,860), equating to 982.23 cases per 10^5^ person-years.

**Fig 2:**
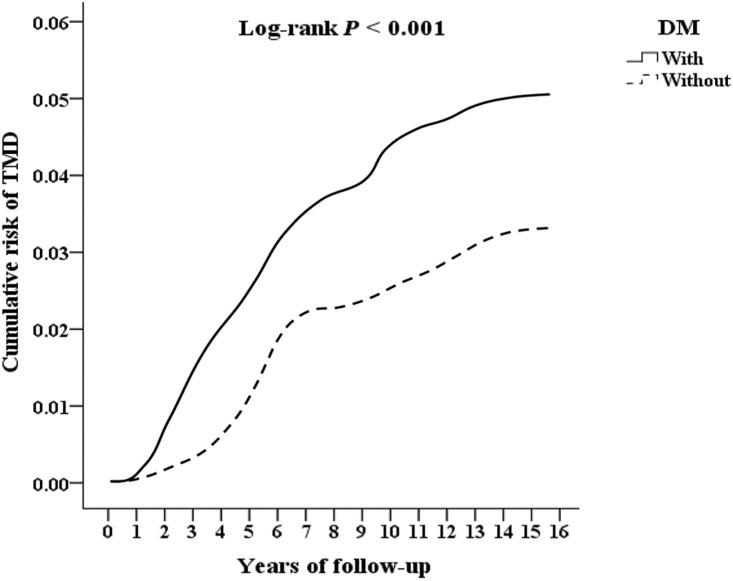
Kaplan–Meier curve of the cumulative risk of temporomandibular disorder stratified by diabetes mellitus, with log-rank test results.

The Kaplan–Meier method revealed a significant difference between the two groups (log rank test: *p* < 0.001; Fig 2). In other words, individuals with DM exhibited a significantly greater risk of developing TMD compared to those without DM. Both cohorts had an average follow-up duration of 8.78 years (see S2 Table). However, the mean time to TMD diagnosis was shorter in the DM group, averaging 5.05 years, compared to 6.01 years in the control group (see S3 Table).

Table 1 presents the baseline characteristics and comorbidity profiles of both the DM and control groups. No significant differences were observed between the two groups in terms of age, sex, and certain comorbid conditions, including rheumatoid arthritis, lower back pain, systemic lupus erythematosus, Sjögren’s syndrome, dermatomyositis, polymyositis, ulcerative colitis, biliary cirrhosis, and Hashimoto’s thyroiditis. However, statistically significant differences were found for geographic location (*p* < 0.001), urbanization level (*p* < 0.001), level of healthcare facility (*p* < 0.001), and insurance premium tier (*p* < 0.001). Additionally, disparities were noted in several medical conditions, including hypertension (*p* < 0.001), ischemic heart disease (*p* < 0.001), chronic obstructive pulmonary disease (*p* < 0.001), cerebrovascular accident (*p* < 0.001), chronic renal insufficiency (*p* < 0.001), depression (*p* < 0.001), ankylosing spondylitis (*p* < 0.05), tinnitus (*p* < 0.001), obstructive sleep apnea (*p* < 0.001), alopecia areata (*p* < 0.05), and Graves’ disease (*p* < 0.001) between the two groups.

**Table 1:** Baseline characteristics of the patients enrolled in the study.

Variables/Group	Total-N (%)	DM-N (%)	Non-DM-N (%)
**Total**	1,007,325	201,465 (20)	805,860 (80)
**Gender**			
Male	602,735 (59.84)	120,547 (59.84)	482,188 (59.84)
Female	404,590 (40.16)	80,918 (40.16)	323,672 (40.16)
**Age** (years)	61.16±19.86	61.14 ± 19.84	61.17 ± 19.86
**Age**			
20-29	8,265 (0.82)	1,653 (0.82)	6,612 (0.82)
30-39	51,205 (5.08)	10,241 (5.08)	40,964 (5.08)
40-49	120,620 (11.97)	24,124 (11.97)	96,496 (11.97)
50-59	200,605 (19.91)	40,121 (19.91)	160,484 (19.91)
60-69	226,230 (22.46)	45,246 (22.46)	180,984 (22.46)
≧70	210,505 (18.85)	42,101 (18.85)	168,404 (18.85)
**Insured premium (NT$)**			
<18,000	631,569 (62.70)	128,972 (64.02)	502,597 (62.37)
18,000-34,999	232,334 (23.06)	45,701 (22.68)	186,633 (23.16)
≧35,000	143,422 (14.24)	26,792 (13.30)	116,630 (14.47)
**Hypertension**	263,594 (26.17)	62,143 (30.85) **	201,451 (25.00)
**Ischemic heart disease**	168,061 (16.68)	43,501 (21.59) **	124,560 (15.46)
**COPD**	135,272 (13.43)	25,018 (12.42) **	110,254 (13.68)
**CVA**	113,547 (11.27)	23,212 (11.52) **	90,335 (11.21)
**CRI**	114,834 (11.40)	24,797 (12.31) **	90,037 (11.17)
**Rheumatoid arthritis**	11,280 (1.12)	2,301 (1.14)	8,979 (1.11)
**Depression**	232,931 (18.78)	49,765 (19.81) **	183,166 (18.52)
**Ankylosis spondylitis**	9,704 (0.96)	2,020 (1.00) *	7,684 (0.95)
**Tinnitus**	11,592 (1.15)	2,457 (1.22) **	9,135 (1.13)
**Lower back pain**	10,264 (1.02)	2,032 (1.00)	8,241 (1.02)
**OSA**	28,701 (2.85)	6,524 (3.24) **	22,177 (2.75)
**SLE**	129 (0.01)	28 (0.01)	101 (0.01)
**Sjögren’s Syndrome**	3,780 (0.38)	797 (0.40)	2,983 (0.37)
**Systemic sclerosis**	36 (0.00)	9 (0.00)	27 (0.00)
**Dermatomyositis**	9 (0.00)	2 (0.00)	7 (0.00)
**Polymyositis**	5 (0.00)	1 (0.00)	4 (0.00)
**Alopecia areata**	7,049 (0.95)	1,456 (1.05) *	5,593 (0.93)
**Ulcerative colitis**	969 (0.13)	183 (0.13)	786 (0.13)
**Biliary cirrhosis**	66 (0.01)	14 (0.01)	52 (0.01)
**Graves’ Disease**	747 (0.10)	168 (0.12) **	579 (0.10)
**Hashimoto’s thyroiditis**	713 (0.07)	153 (0.08)	560 (0.07)
**Location (Taiwan)**			
Northern	282,772 (28.07)	57,986 (28.78) **	224,786 (27.89)
Central	261,877 (26.00)	56,743 (28.17) **	205,134 (25.46)
Southern	244,546 (24.28)	56,894 (28.24) **	187,652 (23.29)
Eastern	149,538 (14.85)	25,971 (12.89) **	123,567 (15.33)
Outlying islands	68,592 (6.81)	3,871 (1.92) **	64,721 (8.03)
**Urbanization level**			
1 (Highest)	269,122 (26.72)	55,324 (27.46) **	213,798 (26.53)
2	309,662 (30.74)	59,786 (29.68) **	249,876 (31.01)
3	205,028 (20.35)	42,014 (20.85) **	163,014 (20.23)
4 (Lowest)	223,513 (22.19)	44,341 (22.01) **	179,172 (22.23)
**Level of care**			
Hospital center	327,927 (32.55)	65,789 (32.66) **	262,138 (32.53)
Regional hospital	354,597 (35.20)	79,896 (39.66) **	274,701 (34.09)
Local hospital	324,801 (32.24)	55,780 (27.69) **	269,021 (33.38)

Notes:

Chi-square/Fisher exact test on category variables and t-test on continue variables.

*The pair of subsets showed a significant difference at *p* < 0.05; ** The pair of subsets showed a significant difference at *p* < 0.001.

Abbreviations: DM: diabetes mellitus; COPD: chronic obstructive pulmonary disease; CVA: cerebrovascular accident; CRI: chronic renal insufficiency; OSA: obstructive sleep apnea, SLE: systemic lupus erythematosus.

Table 2 outlines the risk factors for temporomandibular disorder (TMD) identified through Cox regression analysis. The unadjusted hazard ratio (HR) for individuals with diabetes mellitus (DM) was 3.278 (95% CI: 2.298–4.310; *p* < 0.001). After adjusting for covariates, the HR remained significantly elevated at 2.543 (95% CI: 1.786–3.290; *p* < 0.001). Several variables were significantly associated with an increased risk of developing TMD, including age groups 21–40 years (*p* < 0.001), 41–50 years (*p* < 0.001), 51–60 years (*p* < 0.001), 61–70 years (*p* < 0.001), 71–80 years (*p* < 0.001), and >80 years (*p* < 0.001); hypertension *p* < 0.001); ischemic heart disease (*p* < 0.001); chronic obstructive pulmonary disease (*p* < 0.001); cerebrovascular accident (*p* < 0.001); chronic renal insufficiency (*p* < 0.001); rheumatoid arthritis (*p* < 0.001); depression (*p* < 0.001); ankylosing spondylitis (*p* < 0.001); tinnitus (*p* < 0.001); lower back pain (*p* < 0.001); systemic lupus erythematosus (*p* < 0.001); Sjögren’s syndrome (*p* < 0.001), ulcerative colitis (*p* < 0.001); biliary cirrhosis (*p* < 0.001); and Graves’ disease (*p* < 0.001). Additionally, the HRs for developing TMD were found to rise with higher levels of healthcare facility and greater degrees of urbanization. Among the comorbid conditions linked to diabetes mellitus, rheumatoid arthritis exhibited the strongest association with the subsequent development of TMD (aHR: 4.289; 95% CI: 2.974–5.678; *p* < 0.001), followed by ankylosing spondylitis, which also presented a notably elevated risk (aHR: 4.021; 95% CI: 2.658–5.106; *p* < 0.001). To ensure the validity of these findings, diagnostic and sensitivity analyses was performed and shown as S4 Table. The Schoenfeld residual test confirmed that the proportional hazards assumption was met (*p* = 0.813), as illustrated by the horizontal distribution of residuals in Figure S2. Furthermore, Fine & Gray’s competing risk model yielded results consistent with our primary analysis (adjusted sHR: 2.220; *p* < 0.001), demonstrating the robustness of the association between DM and TMD risk.

**Table 2:** Factors associated with TMD in the Cox regression analysis.

Variables/Group	DM vs. Non-DM	
	Crude HR (95% CI)	Adjusted HR (95% CI)
**DM** (vs Non-DM)	3.278 (2.298–4.310) **	2.543 (1.786–3.290) **
**Male** (vs Female)	0.673 (0.489–0.814) **	0.735 (0.572–0.924) **
**Age** (vs ≦ 20)		
20-29	3.279 (2.101–4.012) **	2.765 (1.453–3.584) **
30-39	3.897 (2.784–4.831) **	3.101 (2.174–4.001) **
40-49	2.903 (1.925–3.786) **	2.243 (1.189–3.012) **
50-59	2.845 (1.897–3.708) **	2.189 (1.104–2.876) **
60-69	2.730 (1.830–3.676) **	2.065 (1.088–2.717) **
≧70	2.611 (1.784–3.567) **	1.977 (1.977–2.650) **
**Insured premium** (vs <NT$18,000)		
18,000-34,999	0.867 (0.472–1.057)	0.952 (0.506–1.143)
**Hypertension** (vs without)	2.896 (1.794–3.245) **	2.601 (1.714–3.020) **
**Ischemic heart disease** (vs without)	2.454 (1.678–2.913) **	2.257 (1.526–2.801) **
**COPD** (vs without)	1.988 (1.289–2.706) **	1.762 (1.099–2.580) **
**CVA** (vs without)	2.579 (1.752–2.986) **	2.303 (1.589–2.863) **
**CRI** (vs without)	2.323 (1.650–2.897) **	2.110 (1.435–2.724) **
**Rheumatoid arthritis** (vs without)	4.596 (3.042–5.783) **	4.289 (2.974–5.678) **
**Depression** (vs without)	3.059 (2.562–4.980) **	2.997 (1.795–3.045) **
**Ankylosis spondylitis** (vs without)	4.120 (2.795–5.562) **	4.021 (2.658–5.106) **
**Tinnitus** (vs without)	1.798 (0.789–2.793)	1.533 (0.594–2.596)
**Lower back pain** (vs without)	1.453 (1.099–1.567) *	1.384 (1.010–1.522) *
**OSA** (vs without)	1.896 (1.232–2.403) **	1.672 (1.085–2.274) *
**SLE** (vs without)	3.379 (2.159–4.303) **	2.978 (1.634–3.850) **
**Sjögren’s Syndrome** (vs without)	3.712 (2.274–4.598) **	3.121 (1.896–3.927) **
**Systemic sclerosis** (vs without)	0.000	0.000
**Dermatomyositis** (vs without)	0.000	0.000
**Polymyositis** (vs without)	0.000	0.000
**Alopecia areata** (vs without)	1.453 (0.978–1.980)	1.203 (0.778–1.763)
**Ulcerative colitis** (vs without)	1.629 (1.129–2.010) **	1.530 (1.035–1.978) *
**Biliary cirrhosis** (vs without)	1.684 (1.186–2.043) **	1.554 (1.053–1.999) *
**Graves’ Disease** (vs without)	1.603 (1.104–1.997) **	1.497 (1.023–1.902) *
**Hashimoto’s thyroiditis** (vs without)	1.578 (1.088–1.983) *	1.428 (1.006–1.894) *
**Location** (vs Northern Taiwan)		
Central	0.974 (0.622–1.277)	Multicollinearity with urbanization level
Southern	1.065 (0.786–1.450)	
Eastern	0.782 (0.433–1.021)	
Outlying islands	0.974 (0.318–0.976)	
**Urbanization level** (vs level 4, lowest)		
1 (Highest)	2.268 (1.796–2.896) **	2.083 (1.591–2.683) **
2	2.011 (1.589–2.482) **	1.825 (1.311–2.227) **
3	1.584 (1.074–1.963)	1.330 (0.892–1.780)
**Level of care** (vs Local hospital)		
Hospital center	0.683 (0.486–0.961)	0.573 (0.384–0.855)
Regional hospital	0.684 (0.495–0.945)	0.697 (0.500–0.973)

Notes:

Adjusted HR: Adjusted variables listed in the table.

*The pair of subsets showed a significant difference at *p <* 0.05; ** The pair of subsets showed a significant difference at *p <* 0.001.

Abbreviations: HR = hazard ratio; CI = confidence interval; DM: diabetes mellitus; COPD: chronic obstructive pulmonary disease; CVA: cerebrovascular accident; CRI: chronic renal insufficiency; OSA: obstructive sleep apnea, SLE: systemic lupus erythematosus.

Table 3 shows that all DM types were significantly associated with TMD compared to the control group (*p* < 0.01). After adjusting for factors such as sex, age, insurance level, comorbid conditions, season, urbanization, and healthcare facility tier, the adjusted HRs for the relationship between TMD and various types of diabetes were as follows: 2.543 (95% CI: 1.786–3.290; *p* < 0.001) for type 1 DM, 1.643 (95% CI: 1.154–2.119; *p* < 0.001) for type 2 DM, 2.603 (95% CI: 2.827–3.364; *p* < 0.001) for other specified types, and 1.551 (95% CI: 1.090–2.008; *p* < 0.001) for unspecified types.

**Table 3:** Factors of TMD among different DM subgroups by using Cox regression.

DM	Populations	Events	Adjusted HR (95% CI)
**Without**	805,860	25,790	Reference
**With DM (Total)**	201,465	10,124	**2.543** (1.786–3.290) **
**T1DM**	7,805	253	1.643 (1.154–2.119) **
**T2DM**	189,763	9,756	**2.603** (1.827–3.364) **
**Other DM**	3,897	115	1.551 (1.090–2.008) *

Notes:

Adjusted HR: Adjusted variables listed in the table.

*The pair of subsets showed a significant difference at *p <* 0.05; ** The pair of subsets showed a significant difference at *p <* 0.001.

Abbreviations: PYs: Person-years; Rate: per 100,000 PYs; HR: hazard ratio, CI: confidence interval.

## Discussion

To our knowledge, this study is the first to examine the risk of TMD in a nationwide, population-based cohort of individuals with DM. The findings indicate that patients with DM had a significantly higher incidence of developing TMD compared to matched controls. Even after adjusting for various covariates, DM remained a significant risk factor for subsequent TMD. The findings of this nationwide cohort study demonstrate a significant association between DM and an increased risk of TMD, with an adjusted HR of 2.543. This aligns with previous smaller-scale clinical observations suggesting that metabolic dysregulation may adversely affect the temporomandibular joint [28]. Compared to earlier studies which often relied on localized clinical samples, our research utilizes a large-scale database (LGTD), providing evidence on Taiwan population [29,30]. While some previous research focused primarily on joint mobility, our study encompasses a broader spectrum of TMD diagnoses, highlighting that the impact of DM may be more pervasive across different TMD manifestations [31]. Specifically, the notably high risk observed in DM patients with comorbid RA or AS suggests a synergistic effect of systemic inflammation on the degradation of the masticatory system [32,33]. This underscores the necessity for multi-disciplinary collaboration between endocrinologists and dental specialists for early screening and intervention. Prior research has suggested that individuals with DM and additional comorbidities are more prone to developing joint-related conditions. The current results are consistent with those reported in earlier studies [15,34–38].

The pathogenesis of the impact of DM on TMD remains uncertain, but some evidence suggests that the damaging effects of advanced glycation end products on connective tissue may be responsible for joint disorders [15,39]. DM can potentially trigger the progression of other inflammatory diseases such as rheumatoid arthritis [40]. In addition, patients with DM are prone to comorbidities such as ankylosing spondylitis and lower back pain [34,36,38,40–42].

A recent animal study involving diabetic rats showed significant morphological alterations in the TMJ, including a notable thinning of the capillaries in the retro-discal tissue and a reduction in the diameter of the articular disc [43]. A subsequent study identified peripheral diabetic neuropathy as an independent risk factor for TMJ dysfunction among individuals with diabetes [16]. These results imply a potential link between TMD and DM, possibly mediated by microvascular complications [15].

Rheumatoid arthritis and ankylosing spondylitis are associated with more than a fourfold increase in the risk of TMD in DM patients, with an aHR of 4.289 and 4.021, respectively. These results align with prior research and are supported by previously reported statistical data [38,42]. Rheumatoid arthritis and ankylosing spondylitis are the highest and second-highest risk factors of TMD among the comorbidities of DM mentioned in this study. Our findings are further strengthened by the sensitivity analysis (S4 Table), which demonstrated that even after accounting for all-cause mortality, patients with DM faced a significantly higher risk of TMD (sHR: 2.220; 95% CI: 1.207–2.935; *p* < 0.001). This consistency between models confirms the stability of DM as an independent risk factor. Furthermore, the study explored the role of autoimmune conditions like rheumatoid arthritis and ankylosing spondylitis. While these conditions exhibited the highest hazard ratios for TMD in our cohort, the persistent significance of DM in models excluding these variables (S4 Table, Model 2) suggests a direct or alternative metabolic pathway contributing to TMD. Chronic pain associated with TMD has been shown to adversely impact overall quality of life [44]. Sleep disruption caused by pain from TMD can contribute to the development of sleep apnea and insomnia. Additionally, the discomfort associated with TMD can significantly impact life satisfaction, which is more commonly observed in patients with DM [45,46].

### Strengths of this study

This study offers important insights for both clinical practice and future research. Utilizing the LGTD presents several strengths, such as a substantial sample size, extended follow-up duration, and reduced selection bias during participant enrollment. The findings revealed a significant association between DM and an elevated risk of developing TMD. Moreover, the signs of TMD may emerge during the follow-up period, ranging from less than a year to more than 15 years, after the patients are diagnosed with DM. While the exact causal relationship has yet to be fully established, this study contributes to a better understanding of the strength and direction of the association between DM and TMD, offering a foundation for future investigations into potential mechanisms and therapeutic approaches.

### Limitations of this study

This study has several limitations. First, diagnoses were derived from physician-reported ICD-9-CM codes, which may not be as accurate as diagnoses obtained through direct clinical interviews. To enhance diagnostic reliability, only patients with at least three consecutive outpatient DM diagnoses assigned by an endocrinologist were included. Second, since the study population consisted of individuals who actively sought medical care, the true incidence of disease may have been underestimated. Third, although adjustments were made for known risk factors, certain variables—such as lifestyle habits, dietary patterns, stress levels, body mass index (BMI), and specific clinical laboratory data (e.g., HbA1c levels and duration of diabetes)—were not available in the LGTD and therefore could not be controlled. Crucially, the LGTD lacks detailed data on disease duration and clinical severity. A longer disease duration or greater severity may progressively worsen microvascular complications and partly explain the higher TMD risk, thus the observed association must be interpreted with caution. The lack of these clinical indicators prevents us from analyzing the correlation between the degree of glycemic control and TMD severity. Lastly, the ICD-9-CM coding system includes only a single code for TMD, which encompasses both joint dysfunction and masticatory pain, limiting diagnostic specificity. Hence, we were unable to classify individuals with TMD into a joint subgroup and a mastication subgroup. Forth, while we conducted a sensitivity analysis using Fine & Gray's competing risk model to account for the potential bias (S4 Table), yielding results consistent with our primary model (adjusted sHR: 2.220; *p* < 0.001), the retros*p*ective nature of this cohort study means we can only demonstrate a strong association between DM and TMD rather than definitive causality. Lastly, the ICD-9-CM coding system includes only a single code for TMD, which encompasses both joint dysfunction and masticatory pain, limiting diagnostic specificity. Hence, we were unable to classify individuals with TMD into a joint subgroup and a mastication subgroup. Finally, the results of our study could only demonstrate a strong relationship between DM and TMD.

## Conclusion

This population-based cohort study demonstrates that individuals with DM are significantly associated with a 2.543-fold increased risk of developing TMD compared to those without DM. This association remained robust even after accounting for all-cause mortality as a competing event (adjusted sHR: 2.220; 95% CI: 1.207–2.935). Among DM patients, rheumatoid arthritis and ankylosing spondylitis were identified as the top two comorbidities associated with the highest cumulative risk for TMD development. These results provide crucial indications for dentists, endocrinologists, and rheumatologists to collaborate in the early clinical screening and multidisciplinary management of TMD in patients with DM to improve their overall quality of life.

## Supporting information

S1 Fig Directed Acyclic Graph (DAG) illustrating the hypothesized causal framework for the association between diabetes mellitus (DM) and temporomandibular disorder (TMD). (DOCX)

S2 Fig Schoenfeld residual plot for the assessment of proportional hazards assumption. (DOCX)

S1 Table ICD-9-CM codes for diabetes mellitus, temporomandibular disorder, and comorbidities. (DOCX)

S2 Table Years of follow-up. (DOCX)

S3 Table Time (years) elapsed from diagnosing diabetes mellitus (DM) to diagnosing temporomandibular disorder. (DOCX)

S4 Table Factors of TMD by using Cox regression with / without Fine & Gray's competing risk model. (DOCX)
