# Supplementary material for: Association between diabetes mellitus and the risk of temporomandibular disorder: A nationwide population-based study
Source: PLoS One. 2026 Aug 13;21(8):e0355792. doi: 10.1371/journal.pone.0355792 (PMC13472446; doi:10.1371/journal.pone.0355792)
Supplement: Supplementary file 1 [file pone.0355792.s001.docx]

**S1 Fig.** **Directed Acyclic Graph (DAG) illustrating the hypothesized causal framework for the association between diabetes mellitus (DM) and temporomandibular disorder (TMD).** The DAG outlines the structural relationships used for covariate selection. DM is the primary exposure and TMD is the outcome of interest. Potential confounders (e.g., age, sex, urbanization level, and insurance premium level) are represented as variables affecting both exposure and outcome. Potential mediators (e.g., rheumatoid arthritis and ankylosing spondylitis) are identified on the causal path between DM and TMD. This framework guided the stepwise adjustment strategy and the sensitivity analysis used to isolate the independent effect of DM.
