# Supplementary material for: Association between diabetes mellitus and the risk of temporomandibular disorder: A nationwide population-based study
Source: PLoS One. 2026 Aug 13;21(8):e0355792. doi: 10.1371/journal.pone.0355792 (PMC13472446; doi:10.1371/journal.pone.0355792)
Supplement: Supplementary file 2 [file pone.0355792.s002.docx]

**S2 Fig.** **Schoenfeld residual plot for the assessment of proportional hazards assumption.** The plot displays the scaled Schoenfeld residuals against follow-up time for the primary exposure variable, diabetes mellitus (DM), in the Cox proportional hazards model for the risk of temporomandibular disorder (TMD). The solid line represents the smoothing spline, and the dashed lines indicate the 95% confidence intervals. The lack of a clear trend and a non-significant Global test (*p*=0.813) indicate that the PH assumption was not violated.
