# Supplementary material for: Association between diabetes mellitus and the risk of temporomandibular disorder: A nationwide population-based study
Source: PLoS One. 2026 Aug 13;21(8):e0355792. doi: 10.1371/journal.pone.0355792 (PMC13472446; doi:10.1371/journal.pone.0355792)
Supplement: Supplementary file 3 [file pone.0355792.s003.docx]

**S1 Table.** ICD-9-CM codes for diabetes mellitus, temporomandibular disorder, and comorbidities.

|  | **ICD-9-CM/Definition** |
| --- | --- |
| **Study population:** Diabetes mellitus | 250, 357.2, 362.0, 366.41; and diagnosed by doctors with metabolism specialty; ≧ 3 outpatient visits or 1 inpatient visit |
| **Events:** Temporomandibular disorder (TMD) | 524.6; diagnosed by dentists |
| **Comorbidities** | 1 year before index date |
| Hypertension (HTN) | 362.11, 401-405, 437.2 |
| Ischemic heart disease (ICH) | 410-414 |
| Chronic obstructive pulmonary disease (COPD) | 490-496 |
| Cerebrovascular accident | 430-438 |
| Chronic renal insufficiency | 585 |
| Rheumatoid arthritis (RA) | 714.0 |
| Depression | 296.2, 296.3, 300.4, 311 |
| Ankylosis spondylitis | 720.0 |
| Tinnitus | 388.3 |
| Lower back pain (LBP) | 720.2, 721.3, 721.42, 722.10, 722.52, 722.73, 722.9,v 724.02, 724.2, 724.3, 724.5, 724.9, 847.2 |
| Obstructive sleep apnea (OSA) | 780.51, 780.53, 780.57 |
| Systemic lupus erythematosus (SLE) | 710.0 |
| Sjögren’s syndrome | 710.2 |
| Systemic sclerosis (SS) | 710.1 |
| Dermatomyositis | 710.3 |
| Polymyositis | 710.4 |
| Alopecia areata | 704.01 |
| Ulcerative colitis | 556 |
| Biliary cirrhosis | 571.6 |
| **Autoimmune thyroid diseases** |  |
| Graves’ disease | 242 |
| Hashimoto’s thyroiditis | 245.2 |
