# Supplementary material for: Association between diabetes mellitus and the risk of temporomandibular disorder: A nationwide population-based study
Source: PLoS One. 2026 Aug 13;21(8):e0355792. doi: 10.1371/journal.pone.0355792 (PMC13472446; doi:10.1371/journal.pone.0355792)
Supplement: Supplementary file 4 [file pone.0355792.s004.docx]

**S2 Table.** Years of follow-up.

| **DM** | **Min** | **Median** | **Max** | **Mean ± SD** |
| --- | --- | --- | --- | --- |
| With | 0.01 | 7.25 | 15.92 | 8.74 ± 4.52 |
| Without | 0.01 | 7.41 | 15.94 | 8.79 ± 4.56 |
| Total | 0.01 | 7.33 | 15.94 | 8.78 ± 4.55 |
