# Supplementary material for: Association between diabetes mellitus and the risk of temporomandibular disorder: A nationwide population-based study
Source: PLoS One. 2026 Aug 13;21(8):e0355792. doi: 10.1371/journal.pone.0355792 (PMC13472446; doi:10.1371/journal.pone.0355792)
Supplement: Supplementary file 5 [file pone.0355792.s005.docx]

**S3 Table.** Time (years) elapsed from diagnosing diabetes mellitus (DM) to diagnosing temporomandibular disorder.

| **DM** | **Min** | **Median** | **Max** | **Mean ± SD** |
| --- | --- | --- | --- | --- |
| With | 0.54 | 4.98 | 15.81 | 5.05 ± 3.89 |
| Without | 0.66 | 5.79 | 15.90 | 6.01 ± 4.04 |
| Total | 0.54 | 5.34 | 15.90 | 5.82 ± 4.03 |
