# Supplementary material for: Association between diabetes mellitus and the risk of temporomandibular disorder: A nationwide population-based study
Source: PLoS One. 2026 Aug 13;21(8):e0355792. doi: 10.1371/journal.pone.0355792 (PMC13472446; doi:10.1371/journal.pone.0355792)
Supplement: Supplementary file 6 [file pone.0355792.s006.docx]

**S4 Table.** Factors of TMD by using Cox regression with / without Fine & Gray's competing risk model.

|  | **DM vs. Non-DM** (Reference) | | | |  |
| --- | --- | --- | --- | --- | --- |
|  | No competing risk in the model | | Competing risk in the model | | |
|  | Crude HR (95% CI) | *p* | Crude sHR (95% CI) | *p* | |
| **Model 0** | 3.278 (2.298–4.310) | <0.001 | 3.025 (1.987–4.005) | <0.001 | |
|  | Adjusted HR (95% CI) | *p* | Adjusted sHR (95% CI) | *p* | |
| **Model 1** | 3.072 (2.145–4.083) | <0.001 | 2.783 (1.836–3.722) | <0.001 | |
| **Model 2** | 2.653 (1.833–3.657) | <0.001 | 2.301 (1.594–3.340) | <0.001 | |
| **Model 3** | 2.557 (1.590–3.301) | <0.001 | 2.242 (1.228–2.981) | <0.001 | |
| **Model 4** | 2.543 (1.552–3.290) | <0.001 | 2.220 (1.207–2.935) | <0.001 | |

Notes:

Abbreviations: HR=hazard ratio, CI=confidence interval, sHR=sub-distribution hazard ratio

Competing variable: All-cause mortality

Schoenfeld residuals, Global test:

*p=*0.965 (Model 0, No competing), *p=*0.942 (Model 0, competing),

*p=*0.927 (Model 1, No competing), *p=*0.910 (Model 1, competing),

*p=*0.864 (Model 2, No competing), *p=*0.847 (Model 2, competing),

*p=*0.820 (Model 3, No competing), *p=*0.812 (Model 3, competing),

*p=*0.813 (Model 4, No competing), *p=*0.801 (Model 4, competing)

Model 0: Crude

Model 1: Adjusted for gender, and age

Model 2: Adjusted for gender, age, and comorbidities (excluding RA and AS)

Model 3: Adjusted for gender, age, comorbidities (excluding RA and AS), insured premium, urbanization level, and level of care

Model 4: Adjusted for gender, age, comorbidities, insured premium, urbanization level, and level of care
